# Construction of Highly Stable Cytotoxic Nuclear-Directed Ribonucleases

**DOI:** 10.3390/molecules23123273

**Published:** 2018-12-11

**Authors:** David Roura Padrosa, Jessica Castro, Alejandro Romero-Casañas, Marc Ribó, Maria Vilanova, Antoni Benito

**Affiliations:** 1 Laboratori d’Enginyeria de Proteïnes, Departament de Biologia, Facultat de Ciències, Universitat de Girona, Campus de Montilivi, Maria Aurèlia Capmany 40, 17003 Girona, Spain; pcxdr1@exmail.nottingham.ac.uk (D.R.P.); jessica.castro@udg.edu (J.C.); alejandro.romero@udg.edu (A.R.-C.); marc.ribo@udg.edu (M.R.); maria.vilanova@udg.edu (M.V.); 2 Institut d’Investigació Biomèdica de Girona Josep Trueta, (IdIBGi), 17190 Girona, Spain

**Keywords:** antitumor protein, nuclear-directed ribonuclease, disulfide bond, thermal stability, proteolytic resistance

## Abstract

Ribonucleases are proteins whose use is promising in anticancer therapy. We have previously constructed different human pancreatic ribonuclease variants that are selectively cytotoxic for tumor cells by introducing a nuclear localization signal into their sequence. However, these modifications produced an important decrease in their stability compromising their behavior in vivo. Here, we show that we can significantly increase the thermal stability of these cytotoxic proteins by introducing additional disulfide bonds by site-directed mutagenesis. One of these variants increases its thermal stability by around 17 °C, without affecting its catalytic activity while maintaining the cytotoxic activity against tumor cells. We also show that the most stable variant is significantly more resistant to proteolysis when incubated with proteinase K or with human sera, suggesting that its half-live could be increased in vivo once administered.

## 1. Introduction

The increasing use of recombinant proteins for therapeutic purposes has highlighted certain issues, such as the control of the lifetime in the bloodstream or the long-term storage of the drug, that are related to the proteins’ stability. Maintenance of a protein drug’s native structure is not only critical for the specific interactions with the target molecules, but also determines its solubility and its transport in a physiological environment. Along these lines, the production of new proteins with enhanced therapeutic abilities may be hampered by the reduction of the protein’s stability upon introduction of the designed mutations. Using strategies aimed to increase the stability of recombinant proteins is therefore an important issue. Among these strategies, we can mention the introduction of additional disulfide bonds [1], prolines [2], salt bridges [3], or the sequence modification to increase the propensity to adopt a given secondary structure [4].

Ribonucleases (RNases) are promising agents to use in anticancer therapy. RNase cytotoxicity requires interaction with the cell membrane, which is followed by endocytosis. Then, cytotoxic RNases are translocated to the cytosol where they evade the cytosolic RNase inhibitor (RI) and cleave the cellular RNA inducing apoptosis [5,6]. RI is a protein that tightly binds to most pancreatic-type RNases inhibiting them and it is thought to act as a safeguard against extracellular RNases that could accidentally reach the cytosol [7]. We have previously shown that introducing an engineered nuclear localization signal (NLS) into the sequence of the human pancreatic RNase (HP-RNase) endows this enzyme with selective cytotoxic activity against tumor cells. These RNases, called nuclear directed-RNases (ND-RNases), are directed to the nuclear compartment which is free of RI [8]. The first example of this strategy consisted in a cytotoxic HP-RNase variant named PE5 that was able to cleave nuclear RNA, inducing apoptosis in tumor cells and reducing the amount of P-glycoprotein in different multidrug-resistant cell lines [9,10,11,12]. These RNases are inhibited by the RI in vitro [9,13]. The region of the RNase recognized by the NLS-mediated import machinery is also involved in the binding to the RI. Consequently, either the RI or the α-importin can bind to the ND-RNase but not both at the same time. As discussed elsewhere [14], a competence between the RI and the α-importin is stablished inside the cell. In this situation, some ND-RNase molecules could be captured by the α-importin and released into the nucleus. The lack of free ND-RNase molecules in the cytosol would shift the RI:ND-RNase equilibrium toward dissociation releasing free ND-RNase, which could be captured by the importin and released again into the nucleus. The characterization of the gene expression pattern of PE5 treated-cells versus untreated cells has shown that this RNase affects multiple metabolic pathways that are important for cancer development [13]. 

PE5 was derived from PM5 [15], a non-cytotoxic HP-RNase variant that presented a higher thermal stability and was produced at high yields, by introducing a conformational bipartite NLS into its structure [9,10]. However, this modification brought a decrease in the T_1/2_ of around 13 °C. A second generation of ND-RNases was obtained by the addition to PE5 of new NLSs fused at different sites [16]. One of these variants, termed NLSPE5, is ten times more cytotoxic than PE5 but has an even slightly lower thermal stability that compromises its use in vivo. 

To overcome this weakness, NLSPE5 has been engineered to increase its thermal stability by introducing additional disulfide bonds. We have produced a variant that increases its thermal stability by around 17 °C, slightly increasing its catalytic activity and retaining the cytotoxic activity against tumor cells. The increase in stability resulted in a significant resistance to proteolysis when the protein was incubated with proteinase K or with human sera.

## 2. Results and Discussion

### 2.1. Design of ND-RNase Variants with Additional Disulfide Bonds

Thermal stabilization of ND-RNases can ameliorate their anticancer properties for two reasons. First of all, the stabilization should increase their resistance to proteolysis once they are distributed throughout the body. It is widely recognized that proteolytic resistance is dependent on the stability of the protein, increasing alongside its own stability [17,18]. Second, it has been described that the thermal stability of the antitumor RNases is an important factor in their cytotoxicity, likely by limiting their proteolytic degradation once the protein reaches the cytosol [1]. ND-RNases contain four native disulfide bonds that tether the protein molecules, contributing to its thermal stability. The introduction of additional disulfide bonds is an alternative for increasing the thermal stability of the protein (for a review, see [19,20]). 

**Figure 1:**
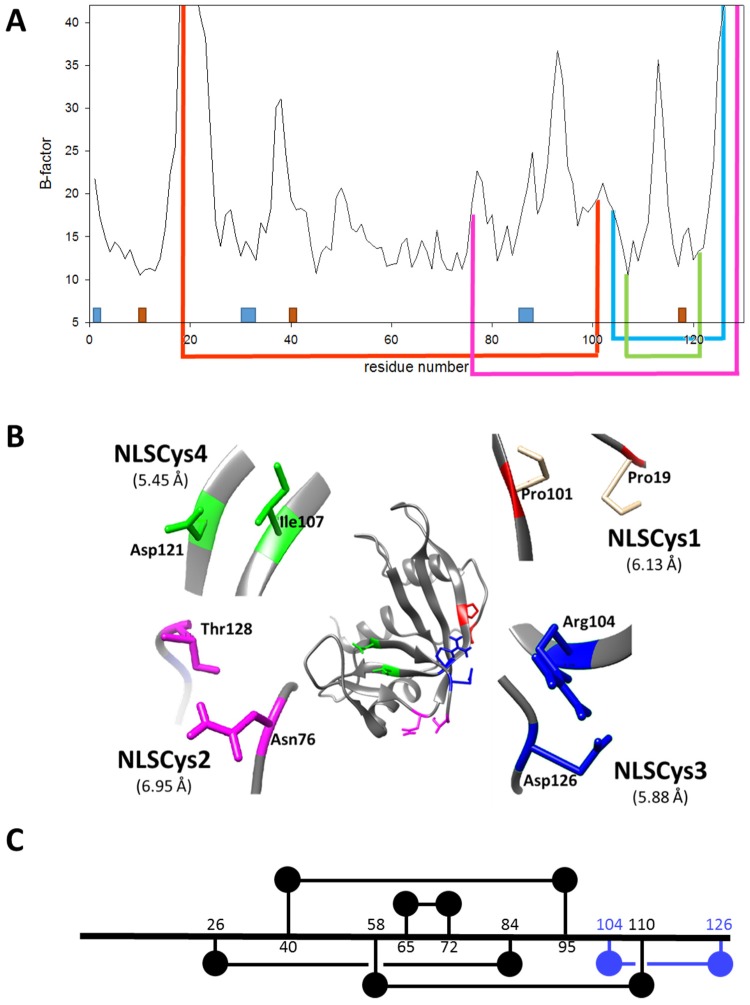
Design of the ND-RNase variants. (**A**) Location of the engineered additional disulfide bonds in the primary sequence of the ND-RNase. B-factor plot for each residue of the sequence (pdb 1dza). Orange boxes show the position of the catalytic triad of the enzyme (His12, His119 and Lys41) and blue boxes those of the residues important for α-importin binding (Lys1, Arg31–33 and Arg89–91). Engineered disulfide bonds are indicated under the plot by lines connecting the created cysteines in NLSPE5Cys1 (red), NLSPE5Cys2 (pink), NLSPE5Cys3 (blue), and NLSPE5Cys4 (green). (**B**) Ribbon diagram of the modeled structure of PE5 ND-RNase and detailed disposition of the different pairs of residues substituted by cysteine in each variant. (**C**) Amino acid connectivity of the five disulfide bonds in the most stable variant NLSPE5Cys3. Black lines correspond to the disulfide bonds already present in the parental RNase and blue line to the new introduced disulfide bond.

Therefore, we aimed to introduce an additional disulfide bond in the ND-RNase NLSPE5. The positions where to introduce cysteine residues in the NLSPE5 sequence in order to create a new disulfide bond were chosen from the analysis of a modeled structure of the ND-RNase according to the following rationale:

(1) In order to be cytotoxic the ND-RNases must interact with α-importin [10], so we excluded those residues placed on the predicted zones of interaction of the ND-RNase with the importin; that is, around the N-terminus of α-helix 1 of the HP-RNase, the C-terminus of α-helix 2 and into the loop connecting β-strands 4 and 5. We also excluded those residues important for the catalytic activity of the RNase (see Figure 1A).
(2) We then inspected the modeled structure to find residue pairs with α-carbons placed at a distance between 5 and 7 Å. This range of distances is slightly higher than that found between the two α-carbon atoms of the disulfide bonds of the modeled structure (between 5.2 and 5.8 Å).
(3) Among the residue pairs following the former criteria, we investigated the relative disposition of their side chains. We only took into account those residue pairs that had side chains facing each other or that were arranged in a parallel way, and we excluded those pairs that were facing away from each other on the structure.
(4) We considered that it was possible the protein would have to rearrange locally when introducing a disulfide bond. Therefore, the introduction of the new disulfide bond between residues located at the most rigid regions of the protein was not a suitable strategy. This idea has been confirmed experimentally [21] and in silico [22]. Dani and colleagues [22] analyzed previously engineered disulfide bonds in a set of recombinant proteins where the effect on protein stability was known and the wild type crystal structure was available. They reported that stabilizing mutations were mostly found in regions of medium to high mobility and near the protein surface in loops larger than 25 residues. Here, we only considered those residue pairs in which at least one of them was placed in a highly mobile region. In the HP-RNase structure, the C-terminal region is highly mobile (Figure 1A). Likewise, the hinge loop connecting the first alpha helix with the rest of the protein (residues 16 to 24) also presents high mobility and indeed in some structures could not be solved (for a review, see [23]). Therefore, residues present in these regions were preferably considered.

According to these criteria, we selected three residue pairs to be replaced by cysteine: Pro19–Pro101, Asn76–Thr128 and Arg104–Asp126. These residues were located at flexible regions along the protein (Figure 1A) as indicated by their B-factors [24]. Their location in the HP-RNase structure, and the distance and orientation of their side chains are shown in Figure 1B. As a proof of concept of the importance of the region’s flexibility where cysteines are introduced, we also selected two residues, Ile107 and Asp121, that despite having their lateral chains arranged in a parallel way and a distance between both alpha-carbons of 5.45 Å (Figure 1B), they were located at positions with low flexibility (Figure 1A). It was expected in this case that the variant displayed a lower thermal stability. All these aforementioned residues were substituted by cysteine through site-directed mutagenesis in order to create the variants NLSPE5Cys1, NLSPE5Cys2, NLSPE5Cys3, and NLSPE5Cys4 (see Table 1). A one-dimensional diagram showing the amino acid connectivity of the five disulfide bonds in NLSPE5Cys3 is shown in Figure 1C. These variants were produced in *E. coli* BL21 (DE3) cells in the form of inclusion bodies, solubilized with guanidinium chloride (Gdm-Cl) and reduced glutathione and then refolded in a buffer containing oxidized glutathione. The refolded sample was purified through a monoS column. This purification step allowed for the separation of the ND-RNase with five disulfide bonds from that fraction of the protein that had a lower number of disulfide bonds since in the latter case the free cysteine residues are forming disulfide bonds with glutathione, introducing an extra negative charge into the molecule [25]. We initially expected that the yield of purified protein of the new variants would be lower than that of the parent protein since the presence of an additional disulfide bond could complicate its refolding. However, the final yield of properly folded proteins per liter of the induced culture was even higher for NLSPE5Cys3 and only lower for NLSPE5Cys4 (Table 1) compared to NLSPE5.

### 2.2. Characterization of the Effect of the Additional Disulfide Bond on the Thermal Stability of the Variants

**Figure 2:**
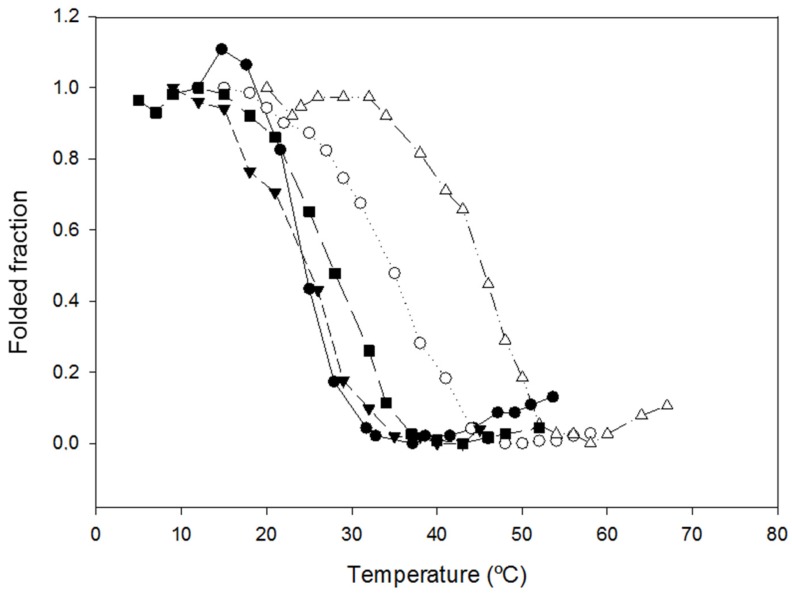
Temperature unfolding curves of the different variants in 1 M Gdm-Cl, 50 mM sodium acetate, pH 4.0. Symbols: NLSPE5 (●); NLSPE5Cys1 (○); NLSPE5Cys2 (▼); NLSPE5Cys3 (Δ); and NLSPE5Cys4 (■). Curves were obtained monitoring the change in absorbance at 280 nm when the temperature was increased.

We investigated the change in thermal stability produced by the introduction of the cysteine residues. We initially carried out these studies by following the unfolding of the protein when increasing the temperature using spectrophotometry. In the first assays, using 50 mM sodium acetate pH 4.0, we observed that the T_1/2_ of NLSPE5Cys2 and NLSPE5Cys4 were very similar to that of the parent protein NLSPE5. However, the post-transition of the denaturation curves of NLSPE5Cys1 and NLSPE5Cys3 could not be observed, although the samples were heated to the highest possible temperature (85 °C). This hampered determining the T_1/2_ and indicated that these two proteins were much more stable than NLSPE5. The experiments were repeated under harsher conditions with the presence of 1 M Gdm-Cl at pH 4.0, and we could obtain the entire single transition-unfolding curve (Figure 2). As shown in Table 1, under these experimental conditions, the T_1/2_ of NLSPE5Cys2 and NLSPE5Cys4 were again very similar to that of NLSPE5, whereas those of NLSPE5Cys1 and NLSPE5Cys3 increased by 16 °C and 27 °C, respectively. We can speculate that the difference in thermal stability between NLSPE5Cys1 and NLSPE5Cys3 could be due in part to the removal of the two proline residues in the former variant. As in the case of the addition of disulfide bonds (see below), the introduction of proline residues decreases the conformational entropy of the denatured state [26]. This is because the rigid pyrrolidine ring constrains the main chain dihedral φ angle leading to the decrease of Cα-N rotation’s conformational freedom in the unfolded state.

For NLSPE5Cys1 and NLSPE5Cys3 and the parent NLSPE5, we also characterized their thermal stability by DSC. This technique allowed for measuring the T_1/2_ in an acetate buffer at pH 5.0 without the presence of the denaturant. For both variants, the unfolding process was completely reversible and thus the thermodynamic parameters could be calculated, and are presented in Table 2. The significant increase in thermal stability was confirmed in the absence of Gdm-Cl although the differences in T_1/2_ respective to NLSPE5 were lower than those previously stated (16.6 °C for NLSPE5Cys3 and 11.1 °C for NLSPE5Cys1). The difference of ΔT_1/2_ respective to that encountered using the spectrophotometric assay could be explained, at least in part, assuming that the effect of Gdm-Cl and a lower pH was higher on the parent variant than on the new variants.

Introducing disulfide bonds is thought to increase the thermal stability by limiting the entropy of the unfolded state, thereby destabilizing the unfolded state respective to the native state [27,28]. In addition, disulfide bonds could increase the thermal stability by increasing the enthalpy of the folded state compared to the unfolded state as a result of burying non-polar groups [29]. However, disulfide bonds can increase or decrease the number of interactions of the folded state that contribute to protein stability; therefore, when engineering new mutants, the effect that a precise disulfide bond may produce in the thermal stability is difficult to predict. For example, in the homologous RNase A, the removal of a single cystine decreases the T_1/2_ by around 20–36 °C [30], but the addition of new cystines has been proven to have a more modest effect on T_1/2_, and in some cases their introduction has even decreased it [31]. More stable variants of RNase A have been created by introducing a cystine between residues 4 and 118 (ΔT_1/2_ of around 5 °C [1]) and between residues 43 and 85 (ΔT_1/2_ of around 2 °C [31]) but the increases in stability are lower than those presented here.

### 2.3. Characterization of the Biological Activities of the Variants

As classically discussed by Shoichet et al., [32], the minimization of protein free energy can produce well-packed hydrophobic interiors and hydrophilic exteriors, but can preclude the formation of the protein active-site, where charged or polar groups are often sequestered from water or hydrophobic patches exposed to solvent. Furthermore, alteration of the sequence of a protein can induce a global rearrangement of the protein structure, altering the active-site cleft. For example, the introduction of a disulfide bond between residues 4 and 118 reduced the catalytic activity of HP-RNase by 10-fold [33] or by 3-fold in the G88R HP-RNase variant [1]. In order to investigate whether the introduction of the cysteine residues had altered the catalytic activity of the ND-RNases, we characterized the kinetic parameters for the hydrolysis of cytidine 2′,3′-cyclic monophosphate (C>p) by NLSPE5 and the constructed variants. Table 3 shows that the kinetic parameters of NLSPE5Cys1 and NLSPE5Cys2 are very similar to those of the parent NLSPE5. Surprisingly, NLSPE5Cys3, the variant with the higher stability, increases its catalytic efficiency compared to the parent NLSPE5. Finally, the catalytic activity and *K*_M_ of NLSPE5Cys4 were clearly altered and thus the catalytic efficiency of this variant is around 12-fold lower than that of the parent variant. It can be postulated that, in this case, the formation of the extra disulfide bond has produced a rearrangement of the protein architecture, which significantly alters the active site conformation.

The cytotoxic activity of the RNases is exerted through a multi-step mechanism (for a review, see [5]) in which the RNase must not only be catalytically active but must also be able to cross the cellular membrane, evade the cytosolic RNase inhibitor and resist proteolysis while it is endocytosed. Therefore, maintenance of ribonucleolytic activity in the constructed variants did not necessarily mean that they had maintained their cytotoxic activity since other parameters could have been altered due to the residue substitutions. Hence, we investigated the effect of the mutations on the cytotoxic activities of the variants respective to NLSPE5 on two different human cell lines: NCI-H460 lung cancer cell line and OVCAR-8 ovarian cancer cell line. In accordance with their catalytic activity parameters, NLSPE5Cys4 was between 30 and 45-fold less cytotoxic than the parent variant whereas the rest of the variants displayed the same cytotoxic activity as NLSPE5 (Table 4). The effect of the different ND-RNases was similar in both cell lines. 

### 2.4. Resistance of the Variants to Proteolysis

Proteolytic degradation of protein-based drugs is seen as a major weakness limiting their therapeutic application. Since protein stabilization and resistance to proteolysis are intimately related, we investigated the effect of proteases on the variants compared to NLSPE5. 

**Figure 3:**
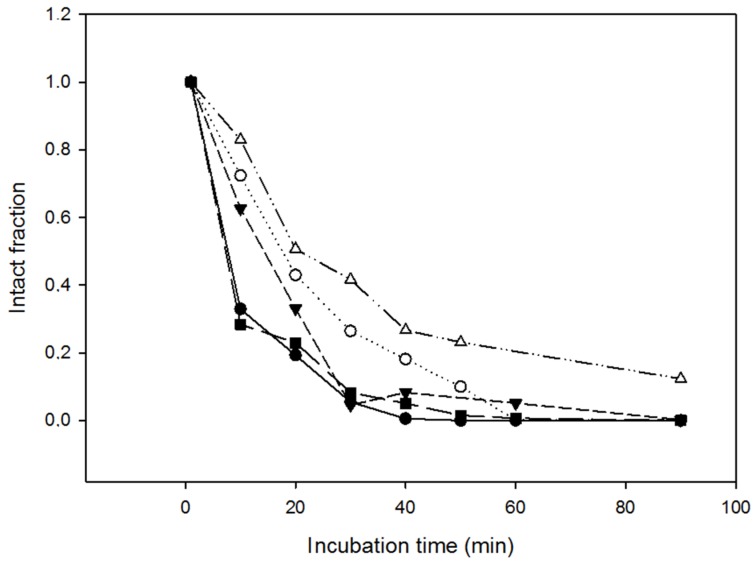
Resistance of the different ND-RNase variants to proteinase K. Amount of intact protein incubated in the presence of proteinase K at 37 °C throughout the incubation period. Symbols: NLSPE5 (●); NLSPE5Cys1 (○); NLSPE5Cys2 (▼); NLSPE5Cys3 (Δ); and NLSPE5Cys4 (■).

Resistance to proteolysis was first checked by incubating the different variants with proteinase K at 37 °C for over 90 min. Samples were withdrawn at varying times, analyzed through SDS-PAGE and the percentage of intact protein was quantified respective to the initial state (Figure 3). This allowed us to measure the half-life of the protein in the reaction, that is, the time required to cleave 50% of the ND-RNase molecules, which corresponded to around 7.5 min for NLSPE5 and NLSPE5Cys4, 14.5 min for NLSPE5Cys2, 18.2 min for NLSPE5Cys1 and 21 min for NLSPE5Cys3.

**Figure 4:**
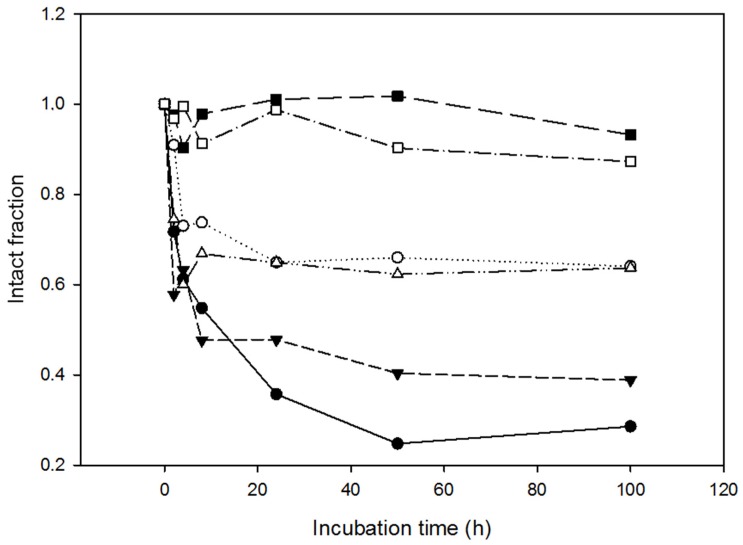
Resistance to proteolysis of the most stable ND-RNase variants when incubated in human sera. Amount of intact NLSPE5 ((● for serum 1 and ○ for serum 2); NLSPE5Cys1 (▼ for serum 1 and Δ for serum 2); and NLSPE5Cys3 (☐ for serum 1 and ■ for serum 2) along time when incubated in the presence of two different human sera at 37 °C.

Proteases are ubiquitous constituents of cells, tissues and body fluids. Specifically, the bloodstream contains proteases participating in hemostasis, fibrinolysis, and tissue conversion, among other activities. We also checked the proteolytic resistance of the most resistant variants, NLSPE5Cys1 and NLSPE5Cys3, when incubated with human sera at 37 °C for 92 h. Two different sera from healthy volunteers were used. Again, the percentage of intact protein compared to the initial moment was quantified using densitometric analysis of SDS-PAGE gels (Figure 4). NLSPE5 and NLSPE5Cys1 were partially cleaved after 3–6 h of incubation at 37 °C, and after 100 h of incubation less than 30–60% of the protein remained intact. In contrast, the percentage of intact NLSPE5Cys3 remained high throughout the experiment with both sera and only around 10% of the molecules were cleaved after 100 h. 

In conclusion, we have designed and produced two new antitumor ND-RNases that have an increased resistance to proteolysis without altering either its catalytic activity or its cytotoxicity for tumor cells. NLSPE5Cys3 is the most interesting candidate among them to be analyzed in vivo as an antitumor drug.

## 3. Materials and Methods 

### 3.1. Plasmid Construction

Construction of NLSPE5 has been previously described [16]. The introduction of two additional cysteine residues to create a disulfide bond was carried out by two successive rounds of site-directed mutagenesis using the Quikchange method (Agilent, Santa Clara, CA, USA). Specific oligonucleotides used to create each variant are listed in Supplementary Table S1. All constructs were confirmed by DNA sequencing.

### 3.2. RNase Expression and Purification

NLSPE5 variants were produced in the form of inclusion bodies in *E. coli* BL21 (DE3) cells transformed with the corresponding vector as previously described [15,25]. Briefly, ND-RNases were solubilized from inclusion bodies with Gdm-Cl and reduced glutathione. The ND-RNases were then refolded through drop dilution in the appropriate buffer containing oxidized glutathione and purified through Mono-Scation-exchange chromatography (GE Healthcare, Marlborough, MA, USA). The molecular mass of each variant was confirmed by matrix-assisted laser desorption/ionization time-of-flight mass spectrometry (Bruker, Billerica, MA, USA). The protein concentration of each variant was determined by UV spectroscopy in a Lambda Bio 20 spectrophotometer (PerkinElmer, Waltham, MA, USA), using molar extinction coefficients of 7950 M^−1^ cm^−1^ for NLSPE5 and 8075 M^−1^ cm^−1^ for the variants carrying two additional cysteines. These extinction coefficients were calculated using the method devised by Pace et al. [34]. 

### 3.3. Spectrophotometric Determination of Thermal Stability

Temperature-unfolding studies were essentially carried out as described [35]. Proteins were dissolved to a concentration of 0.8–1 mg/mL in 50 mM sodium acetate, pH 4.0 or in 1 M Gdm-Cl, 50 mM sodium acetate, pH 4.0. Temperature-unfolding was followed spectrophotometrically at 280 nm. Transition curves were fitted to a two-state thermodynamic model combined with sloping linear functions for the native and denatured states, and the thermodynamic parameters were calculated as previously reported [36]. All data are described as the mean ± standard error (SE) of three independent determinations.

### 3.4. Calorimetric Determination of Thermal Stability

Microcalorimetric measurements were carried out on a nano DSC differential scanning microcalorimeter (TA Instruments, New Castle, DE, USA), equipped with a capillary cell, at a heating rate of 1 K/min from 298 to 373 K and a constant pressure of 0.3 MPa. Protein solutions were prepared at 1 mg/mL in 50 mM sodium acetate buffer at pH 5.0. The heating curves were corrected for an instrumental baseline obtained by heating the solvent used for the protein solution. T_1/2_, calorimetric denaturation enthalpy (ΔHcal) and free energy of unfolding (ΔG_T1/2_) were determined as described in [37]. 

### 3.5. Determination of Steady-State Kinetic Parameters

A spectrophotometric assay [38] was used to determine the kinetic parameters of the ND-RNase variants for the hydrolysis of C>p (Sigma, Saint Louis, MO, USA). Steady-state kinetic parameters were obtained from the Lineweaver-Burk plot through regression analysis. All data are described as the mean ± SE of three independent determinations. 

### 3.6. Cell Lines and Culture Conditions

The NCI-H460 human lung cancer cell line and the OVCAR-8 human ovarian cancer cell line were obtained from the National Cancer Institute-Frederick DCTD tumor cell line repository. They were routinely grown at 37 °C in a humidified atmosphere of 5% CO_2_ in RPMI (Gibco, Waltham, MA, USA) supplemented with 10% fetal bovine serum (FBS) (Gibco), 50 U/mL penicillin and 50 µg/mL streptomycin (Gibco). Cells remained free of Mycoplasma and were propagated according to established protocols.

### 3.7. Cell Proliferation Assays

Cells were seeded into 96-well plates at 1500 cells/well for OVCAR-8, and at 1900 for NCI-H460. After 24 h of incubation, cells were treated with various concentrations of RNase for 72 h. Drug sensitivity was determined by the MTT method essentially following the manufacturer’s instructions (Sigma, Saint Louis, MO, USA) and according to [39]. All data are presented as the mean ± SE of at least three independent experiments with three replicates for each.

### 3.8. Resistance to Proteinase K

Sensitivity to proteinase K was investigated essentially as described [40]. All proteins were prepared at a final concentration of 10 µM in 50 mM TrisHCl, 150 mM NaCl pH 8.0 and incubated with proteinase K recombinant PCR grade (Roche, Basel, Switzerland) at a final concentration of 0.35 µM for different times (0 to 90 min) at room temperature. PMSF (1.5 µM) was used to stop the reaction, and samples were then analyzed by SDS-polyacrilamide gel electrophoresis (SDS-PAGE) (15% polyacrylamide). Quantification of the intact protein in the gel was performed with ImageJ software (Wisconsin, MA, USA) [41]. 

### 3.9. Analysis of the Proteolysis Resistance in Serum

A sample of whole blood was drawn at the Dr. Josep Trueta University Hospital of Girona from two healthy human volunteers that had signed the informed consent. The samples were allowed to clot, centrifuged at 2500× *g* for 5 min and the sera were collected in aliquots and frozen at −80 °C, without heat inactivation or cryopreservation, until use. All the variants (10 μM final concentration in PBS) were incubated at 37 °C with an equal volume of the different sera. Aliquots were withdrawn at different times (0 to 100 h), mixed with Laemmli buffer and stored at −20 °C. Samples were analyzed by SDS-PAGE (15% polyacrylamide). Quantification of the intact protein in the gel was performed using Quantity One software (Bio-Rad Laboratories, Hercules, CA, USA). 

## Figures and Tables

**Table 1:** Yield of purified ND-RNase carrying five disulfide bonds and their T_1/2_ calculated using UV-spectrophotometry.

Name	Replacements	Yield ^1^	T_1/2_^2^
NLSPE5	-	15 mg	23.6 °C
NLSPE5Cys1	Pro19–Pro101	15 mg	38.7 °C
NLSPE5Cys2	Asn76–Thr128	15 mg	26.1 °C
NLSPE5Cys3	Arg104–Asp126	30 mg	49.9 °C
NLSPE5Cys4	Ile107–Asp121	3 mg	29.3 °C

^1^ Yield of purified protein per L of induced culture. ^2^ T_1/2_ calculated spectrophotometrically in a buffer containing sodium acetate pH 4.0 and 1 M Gdm-Cl.

**Table 2:** Thermodynamic parameters of the temperature unfolding of the variants at pH 5.0 using Differential Scanning Calorimetry.

	NLSPE5	NLSPE5Cys1	NLSPE5Cys3
T_1/2_ (°C)	48.3 ± 0.4	59.4 ± 0.2	64.9 ± 1.0
ΔHcal (kJ/mol)	241.1 ± 1.6	273.2 ± 0.1	308.3 ± 8.4
ΔΔG (kJ/mol)	-	−8.35 ± 0.2	−12.47 ± 0.35

**Table 3:** Catalytic parameters for the hydrolysis of C>p of the different ND-RNase variants.

Parameter	ND-RNase Variant				
	NLSPE5	NLSPE5Cys1	NLSPE5Cys2	NLSPE5Cys3	NLSPE5Cys4
*K*_cat_ (min^−1^)	413 ± 191	695 ± 131	480 ± 48	573 ± 7	139 ± 41
*K*_M_ (mM)	0.88 ± 0.53	1.34 ± 0.11	1.44 ± 0.27	0.65 ± 0.15	4.08 ± 0.51
Relative *K*_cat_/*K*_M_ (%)	100 ± 7.7	134.8 ± 14.6	88.9 ± 25.4	233.1 ± 49.3	8.8 ± 1.5

**Table 4:** IC_50_ values (µM) of the different ND-RNases on OVCAR-8 and NCI-H460 cell lines.

Cell Line	NLSPE5	NLSPE5Cys1	NLSPE5Cys2	NLSPE5Cys3	NLSPE5Cys4
OVCAR-8	0.2 ± 0.1	0.3 ± 0.2	0.4 ± 0.2	0.2 ± 0.1	6.9 ± 3.8
NCI-H460	0.2 ± 0.1	0.3 ± 0.1	0.2 ± 0.1	0.2 ± 0.1	9.2 ± 4.3

## Supplementary Materials

The following are available online. Table S1: List of oligonucleotides used to introduce the different cysteine residues on the NLSPE5.
