# Supplementary material for: Construction of Highly Stable Cytotoxic Nuclear-Directed Ribonucleases
Source: Molecules. 2018 Dec 11;23(12):3273. doi: 10.3390/molecules23123273 (PMC6321540; doi:10.3390/molecules23123273)
Supplement: Supplementary file 1 [file molecules-23-03273-s001.pdf]

Table S1. List of oligonucleotides used to introduce the different cysteine residues on the NLSPE5 gene.

Substitute d residue	Name	Sequence
Pro19	P19C_1	GGACTCTGGTAACTCCTGTTCGAGCTCTTCTAC
	P19C_2	GTAGAAGAGCTCGAACAGGAGTTACCAGAGTCC
Pro101	P101C_1	GTGCTTACAGAACTTCTTGTAAAGAAAGACACATTATTG
	P101C_2	CAATAATGTGTCTTTCTTTACAAGAAGTTCTGTAAGCAC
Asn76	N76C_1	GGTAATTGTTACAAATCTTGTTCTAGCATGCATATTAC
	N76C_2	GTAATATGCATGCTAGAACAAGATTTGTAACAATTACC
Thr128	T128C_1	GCGTTGAAGATTCTTGTTAAGTCGAGCAGATCCG
	T128C_2	CGGATCTGCTCGACTTAACAAGAATCTTCAACGC
Ile107	I107C_1	CCTAAAGAAAGACACATTTGTGTTGCTTGTGAAGGTTC
	I107C_2	GAACCTTCACAAGCAACACAAATGTGTCTTTCTTTAGG
Asp121	D121C _1	GTTCCTGTTCATTTTTGTGCTAGCGTTGAAGATTC
	D121C _2	GAATCTTCAACGCTAGCACAAAAATGAACAGGAAC
Arg104	R104C_1	GAACTTCTCCTAAAGAATGCCACATTATTGTTGCTTG
	R104C_2	CAAGCAACAATAATGTGGCATTCTTTAGGAGAAGTTC
Asp126	D126C _1	GCTAGCGTTGAATGTTCTACTTAAGTCGAGCAG
	D126C _2	CTGCTCGACTTAAGTAGAACATTCAACGCTAGC
